# The association between patient preferred language and end-of-life outcomes of home care patients who died from a cancer in Ontario, Canada – A retrospective cohort study

**DOI:** 10.1371/journal.pone.0351840

**Published:** 2026-06-22

**Authors:** Tzu-Fei Wang, Lise M. Bjerre, Maya Gibb, Samantha Yoo, Michael Pugliese, WooJin Kim, Aliza Moledina, Franciely Daiana Engel, Wildhine Lominy, Marc Carrier, Peter Tanuseputro, Sharon Johnston, Chantal Backman

**Affiliations:** 1 Department of Medicine, University of Ottawa, Ottawa, Ontario, Canada; 2 Ottawa Hospital Research Institute, Ottawa, Ontario, Canada; 3 ICES uOttawa, Ottawa, Ontario, Canada; 4 Institut du Savoir Montfort, Ottawa, Ontario, Canada; 5 Department of Family Medicine, University of Ottawa, Ottawa, Ontario, Canada; 6 Bruyère Health Research Institute, Ottawa, Ontario, Canada; 7 Faculty of Health Sciences, University of Ottawa, Ottawa, Ontario, Canada; 8 Department of Family Medicine and Primary Care, University of Hong Kong, Hong Kong, Hong Kong; Laval University, CANADA

## Abstract

**Background:**

Language is an often-forgotten determinant of health. The impact of patient preferred language on health outcomes of patients with cancer remains under-investigated.

**Aim:**

To explore the association between patient preferred language and end-of-life outcomes of patients who died of cancer.

**Design/setting/participants:**

We conducted a population-based, retrospective cohort study of home care recipients who died of cancer between 2013 and 2018 in Ontario, Canada. We identified patient preferred language from standardized home care assessments. The primary outcomes included place of death, emergency department (ED) visits and hospital admissions within the last 30 days of life. Secondary outcomes included aggressive interventions. We used descriptive statistics and multivariable logistic regression models to characterize the association between patient language and end-of-life outcomes in this population.

**Results:**

33,958 home care recipients were included, with 28,322 (83.4%) anglophones, 786 (2.3%) francophones, and 4,850 (14.3%) allophones. Allophones were older, more likely to reside in lower-income neighborhoods and more likely to have immigrated to Canada within 5 years. Allophones had significantly higher odds of death in hospital (OR 1.35, 95% CI 1.25–1.45), hospital admissions (OR 1.16, 95% CI 1.07–1.24), ED visits (OR 1.16, 95% CI 1.08–1.24), and increased odds of aggressive interventions in the last 30 days of life compared to anglophones. Francophones also had increased odds of death in hospital (OR 1.23, 95% CI 1.04–1.46) compared to anglophones.

**Conclusion:**

Our study showed that in patients with terminal cancer, allophone home care recipients had more hospital deaths and aggressive interventions compared to anglophones. Additional research is needed to identify root causes and strategies to improve care.

## Introduction

Language is a central component of a person’s identity. In Canada, while English and French are the two official languages, with some variations at the provincial level, linguistic diversity is increasing at an unprecedented rate. Estimates from 2021 suggested that one third of residents live in a province that does not recognize their mother tongue as an official language [1]. As a result, nearly 30% of Ontario’s population, the largest province in Canada, are at risk of experiencing language barriers when accessing healthcare services [1]. Language is a foundational, yet frequently overlooked, determinant of health. Prior research has shown that home care recipients in Ontario who were hospitalized and received the majority of their care from physicians who spoke their primary language had a lower risk of inpatient harms and mortality and shorter length of stays compared to those who received language-discordant care [2].

Cancer is the leading cause of death in Canada [3]. Cancer patients and their families frequently confront complex medical decisions and unique challenges where effective communication is crucial, particularly in discussing complex symptomatology, treatment preferences, goals of care, and end-of-life care. These conversations may be even more challenging when there are language barriers. Despite the recognized importance of communication in oncology, data evaluating the impact of language on patients with cancer remain limited. To address this gap, we conducted a retrospective cohort study in Ontario, Canada, using population-based health administrative data to evaluate the impact of patient preferred language on end-of-life outcomes in home care patients who died of cancer.

## Materials and methods

This project was conducted using databases housed in ICES (formerly the Institute for Clinical Evaluative Science). ICES is a prescribed entity under Sect. 45 of Ontario’s Personal Health Information Protection Act, which authorizes ICES to collect personal health information, without consent, for the purpose of analysis or compiling statistical information with respect to the management of, evaluation or monitoring of, allocation of resources to or planning for all or part of the health system. Projects that use data collected by ICES under Sect. 45 of PHIPA, and use no other data, are exempt from Research Ethics Board (REB) review. The use of the data in this project is authorized under Sect. 45 and was approved by ICES’ Privacy and Legal Office. For this study, databases were accessed between April 2024 and June 2025. The authors had no access to information that could identify individuals in the databases at any time throughout the study. We reported the study using the REporting of studies Conducted using Observational Routinely collected Data (RECORD) guidelines [4].

### Study design and population

We conducted a population-based, retrospective cohort study in Ontario, Canada of home care recipients who died of cancer between January 1, 2013 and December 31, 2018, and who had a Residential Assessment Instrument-Home Care (RAI-HC/interRAI-HC) assessment completed within 2 years of their index date (index date = death date – 180 days). RAI-HC assessments were used to capture the patient’s preferred language and clinical characteristics (such as Activities of Daily Living [ADL], Instrumental Activities of Daily Living [IADL] scales, Changes in Health, End-Stage Disease and Signs and Symptoms [CHESS] Score, Cognitive Performance Scale [CPS], and Pain Scale). At the initiation of home care services, a comprehensive and standardized assessment (RAI-HC) is completed for all patients. For individuals with multiple assessments, we selected the assessment closest to the individuals’ death date. In Ontario, patients are referred for home care services by their healthcare providers when they are determined to have healthcare needs, such as requiring assistance with activities of daily living, medical treatments, therapy services, or palliative care.

We excluded individuals who were: (1) aged less than 18 or more than 105 at index; (2) ineligible for the Ontario Health Insurance Plan (OHIP) in the 2 years preceding the individual’s index date; (3) non-Ontario residents at death date; or (4) had an invalid birth date or gender (data missing from the Registered Persons Database [RPDB]); (5) missing a language assessment in RAI-HC.

### Data sources

We used linked health administrative data from ICES, an independent, non-profit research institute whose legal status under Ontario’s health information privacy law allows it to collect and analyze health care and demographic data, without consent, for health system evaluation and improvement. We obtained data on home care recipients through record-level RAI-HC assessments linked to other health administrative databases housed at ICES. Emergency department visits (ED) and hospital admissions were captured from the National Ambulatory Care Reporting System (NACRS) and the Discharge Abstract Database (DAD), respectively. The Home Care Database (HCD) captured information on palliative home care visits provided by non-physicians while OHIP captured palliative home care visits provided by physicians. Birthdate, sex, postal code, neighbourhood income quintile, and rurality were captured by the RPDB. Date and cause of death (by the type of cancer) were captured from the Office of the Registrar General, Deaths (ORDG) dataset. The Immigration, Refugees and Citizenship Canada (IRCC) Permanent Resident’s Database was used to identify immigrants who became permanent residents after 1985. These datasets were linked using unique encoded identifiers and analyzed at ICES.

### Exposure

We obtained patient preferred language from the RAI-HC assessments, where trained assessors – most often at the patient’s home and in the presence of family/caregivers – determine the home care recipient’s primary language by listening, observing, and asking home care recipients to specify their primary language during interviews covering > 400 items and often lasting for over an hour (https://interrai.org/applications/status-and-outcome-scales/). The primary language variable in the RAI-HC has been previously validated [5]. We categorized home care recipients into three linguistic groups: anglophone (preferred language English), francophone (preferred language French), and allophone (preferred language is any language other than English or French) [1].

### Covariates

We identified a list of pertinent covariates: age, sex, neighbourhood income quintile, rurality, immigration status, region, type of cancer (as cause of death), and Charlson Comorbidity Index. From RAI-HC, we obtained marital status, ADL (lower score indicating lower function), IADL (lower score indicating lower function), CPS (lower score indicating higher cognition), CHESS score (indicating frailty and health instability to identify individuals at high risk of decline, with lower scores indicating better outcomes), and symptoms (pain scale) from the assessment closest to the individual’s death date.

### Outcomes

Our primary outcomes included 1) place of death (in hospital or in the community), 2) ED visits in the last 30 days of life, and 3) hospital admissions in the last 30 days of life. Our secondary outcomes consisted of aggressive interventions in the last 30 days of life including mechanical ventilation, cardiopulmonary resuscitation (CPR), defibrillation, initiation of dialysis, percutaneous coronary intervention (PCI), blood transfusion, bronchoscopy, administration of vasopressors, and insertion of a feeding tube (all identified by International Classification of Diseases [ICD] codes). In addition, admission to an intensive care unit (ICU) in the last 30 days of life and initiation of chemotherapy in the last 14 days of life were evaluated. We also investigated the delivery of palliative care services within the last 180 days of life, including type, location, and duration of palliative care visits prior to death. Please refer to S1 Table for list of interventions and billing codes.

### Statistical Analysis

We performed descriptive statistics to compare the baseline characteristics of patients and outcomes across the three linguistic groups. We evaluated the relationship between our primary and secondary outcomes and home care recipients’ primary language using multivariable logistic regression models, with anglophones as the reference group. In the adjusted analyses, we included potential confounders including age, sex, comorbidity count, neighbourhood income quintile, rurality, immigration status, type of cancer, ADL, CPS, CHESS score, and pain scale. All variables were modelled as categorical except for age and comorbidity count which were fit with restricted cubic splines at the following percentiles: 5, 27.5, 50, 72.5, 95. We used 2-tail statistical tests with significance threshold α of 0.05. SAS Enterprise Guide, version 7.1 (SAS Institute Inc., Cary, NC, USA) was used for analyses.

## Results

### Study cohort

**Fig 1:**
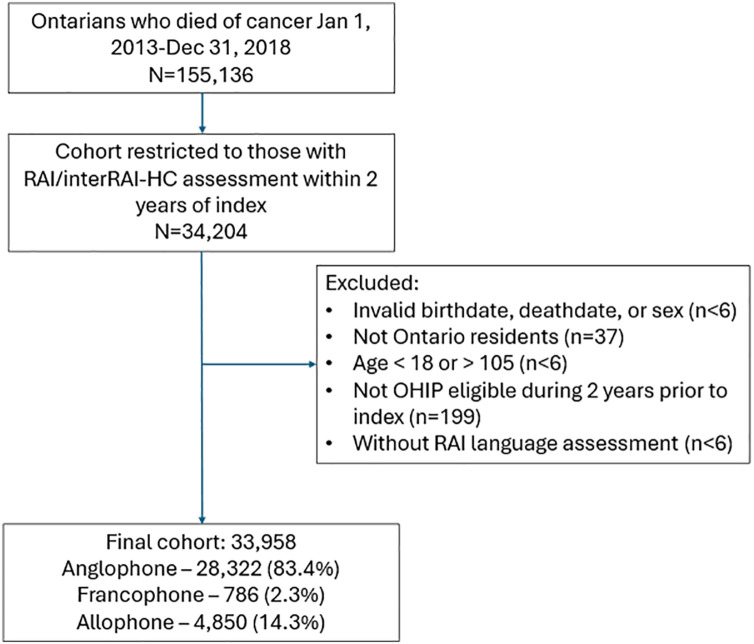
Study flow diagram. RAI-HC: Residential Assessment Instrument-Home Care (RAI-HC/interRAI-HC) a Index = death date – 180 days.

Our study included 33,958 home care recipients, of which 28,322 (83.4%) were anglophone, 786 (2.3%) francophone, and 4,850 (14.3%) allophone (**Fig 1**, **Table 1**). Among allophones, the 10 most spoken languages included: Italian (28.1%), Chinese (12.5%), Portuguese (7.5%), Polish (4.3%), Russian (3.4%), German (3.2%), Greek (3.1%), Arabic (2.7%), Punjabi (2.5%), and Spanish (2.4%).

**Table 1:** Baseline cohort characteristics.

	Total	Anglophone	Francophone	Allophone
	(n = 33,958)	(n = 28,322)	(n = 786)	(n = 4,850)
	N (%)	N (%)	N (%)	N (%)
**Age** (mean, SD)	77.2 (12.6)	76.5 (12.8)	79.7 (10.6)	80.6 (11.1)
**Sex**				
Male	15,663 (46.1)	13,073 (46.2)	359 (45.7)	2,231 (46.0)
Female	18,295 (53.9)	15,249 (53.8)	427 (54.3)	2,619 (54.0)
**Rurality**				
Urban	28,607 (84.2)	23,310 (82.3)	563 (71.6)	4,734 (97.6)
Rural	5,245 (15.4)	4,919 (17.4)	*218-222	*104-108
Missing	106 (0.3)	93 (0.3)	*1-5	*8-12
**Neighbourhood income quintile**				
1 (poorest)	8,498 (25.0)	6,968 (24.6)	217 (27.6)	1,313 (27.1)
2	7,565 (22.3)	6,198 (21.9)	207 (26.3)	1,160 (23.9)
3	6,597 (19.4)	5,497 (19.4)	150 (19.1)	950 (19.6)
4	5,728 (16.9)	4,865 (17.2)	109 (13.9)	754 (15.5)
5 (wealthiest)	5,432 (16.0)	4,676 (16.5)	*98-102	*654-658
Missing	138 (0.4)	118 (0.4)	*1-5	*15-19
**Immigration Status**				
< 5 yrs	85 (0.3)	27 (0.1)	0 (0)	58 (1.2)
5-10 yrs	253 (0.7)	79 (0.3)	*1-5	*169-173
>10 yrs	269 (0.8)	79 (0.3)	*1-5	*185-189
Long term resident	33,351 (98.2)	28,137 (99.3)	*781-785	*4,429−4,433
**Local Health Integration Network**				
North Region	2,856 (8.4)	2,400 (8.5)	293 (37.3)	163 (3.4)
South East Region	2,173 (6.4)	1,669 (5.9)	351 (44.7)	153 (3.2)
South West-Central Region	28,929 (85.2)	24,253 (85.6)	142 (18.1)	4,534 (93.5)
**Marital Status**				
Married/partnered	16,394 (48.3)	13,632 (48.1)	329 (41.9)	2,433 (50.2)
Never married	2,111 (6.2)	1,909 (6.7)	60 (7.6)	142 (2.9)
Separated/divorced	3,382 (10.0)	3,053 (10.8)	67 (8.5)	262 (5.4)
Widowed	12,071 (35.5)	9,728 (34.3)	330 (42.0)	2,013 (41.5)
**Cancer type as Cause of Death**				
Breast	1,701 (5.0)	1,465 (5.2)	34 (4.3)	202 (4.2)
Colorectal	3,163 (9.3)	2,610 (9.2)	74 (9.4)	479 (9.9)
Gastrointestinal (GI) other (excluding colorectal)	2,483 (7.3)	1,955 (6.9)	53 (6.7)	475 (9.8)
Genitourinary (GU) (excluding prostate)	1,573 (4.6)	1,299 (4.6)	45 (5.7)	229 (4.7)
Gynecological	1,331 (3.9)	1,098 (3.9)	27 (3.4)	206 (4.2)
Head and Neck	1,023 (3.0)	854 (3.0)	23 (2.9)	146 (3.0)
Hematological	3,150 (9.3)	2,589 (9.1)	74 (9.4)	487 (10.0)
Lung	5,817 (17.1)	5,012 (17.7)	158 (20.1)	647 (13.3)
Pancreas	1,293 (3.8)	1,042 (3.7)	24 (3.1)	227 (4.7)
Prostate	2,336 (6.9)	1,959 (6.9)	58 (7.4)	319 (6.6)
Other^#^	10,088 (29.7)	8,439 (29.8)	216 (27.5)	1,433 (29.5)
**Prognosis ≤ 6 months^**				
No	31,823 (93.7)	26,466 (93.4)	719 (91.5)	4,638 (95.6)
Yes	2,135 (6.3)	1,856 (6.6)	67 (8.5)	212 (4.4)
**ADL Scale (0–6)**				
Mean (SD)	1.0 (1.4)	1.0 (1.4)	1.2 (1.4)	1.4 (1.6)
Median (Q1-Q3)	0 (0-2)	0 (0-2)	1 (0-2)	1 (0-2)
**IADL Scale (0–6)**				
Mean (SD)	4.1 (1.7)	4.0 (1.7)	4.5 (1.5)	4.6 (1.4)
Median (Q1-Q3)	5 (4–5)	5 (2–5)	5 (4–5)	5 (4–5)
**CHESS Score (0–5)**				
Mean (SD)	1.7 (1.2)	1.7 (1.2)	1.9 (1.2)	1.7 (1.2)
Median (Q1-Q3)	2 (1–2)	2 (1–2)	2 (12–3)	2 (1–2)
**Cognitive Performance Scale (0–6)**				
Mean (SD)	1.2 (1.2)	1.1 (1.2)	1.5 (1.3)	1.5 (1.3)
Median (Q1-Q3)	1 (0-2)	1 (0-2)	2 (0-2)	2 (0-2)
**Pain Scale (0–4)**				
Mean (SD)	1.4 (1.2)	1.4 (1.2)	1.5 (1.1)	1.5 (1.1)
Median (Q1-Q3)	2 (0-2)	2 (0-2)	2 (0-2)	2 (0-2)
**Charleston Comorbidity Index**				
Mean (SD)	4.6 (2.8)	4.6 (2.8)	4.6 (2.9)	4.8 (2.8)
Median (Q1-Q3)	6 (2–7)	6 (2–6)	6 (2–7)	6 (2–7)

**ADL –** Activities for Daily Living (lower score indicating lower function)

**IADL –** Instrumental Activities for Daily Living (lower score indicating lower function)

**CHESS –** Changes in Health, End-Stage Disease and Signs and Symptoms (lower scores indicating better outcomes)

Cognitive Performance Scale – lower score indicating higher cognition

*Cell sizes with < 6 individuals (or if back calculation would lead to another cell size < 6) are suppressed as per ICES policy

# Other cancer types: include cancers in the brain and spine, skin, soft tissue, bone, and metastatic (secondary cancers)

^Defined by a RAI/interRAI variable

### Baseline characteristics

**Table 1** summarizes the baseline characteristics of the cohort. Compared to anglophones (mean [SD] 76.5 [12.8]), francophones (mean [SD] 79.7 [10.6]) and allophones (mean [SD] 80.6 [11.1]) were older. The sex distribution was similar among the linguistic groups, with a greater proportion of the cohort being female (53.9%). The most common type of cancer as the cause of death was lung (17.1%), followed by colorectal (9.3%) and hematological (9.3%). Compared to anglophones, allophones were more likely to reside in urban areas, within lower neighbourhood income quantiles, and more likely to have immigrated within 5 years. Allophones had higher ADL, IADL (higher function) but also higher CPS (lower cognition) and pain scale scores and more comorbidities as determined by Charleston Comorbidity index.

### Outcomes

#### Primary outcomes.

Of the total cohort, 28.2% died in the hospital, while 71.8% died in the community (**Table 2**). The proportion of patients who died in the hospital were highest (32.7%) among allophones, compared to 27.4% among anglophones and 30.5% among francophones. Allophones had the highest rates of hospitalizations (47.8%) and ED visits (52.9%) within the last 30 days of life, compared with 43.5% and 50.1% in anglophones, and 44.1% and 49.4% in francophones, respectively.

**Table 2:** Primary and secondary outcomes.

	Total	Anglophone	Francophone	Allophone	P-value
	(n = 33,958) N (%)	(n = 28,322) N (%)	(n = 786) N (%)	(n = 4,850) N (%)	
**Primary Outcomes**					
**Place of death**					
Community	24,369 (71.8)	20,558 (72.6)	546 (69.5)	3,265 (67.3)	<0.000
Hospital	9,589 (28.2)	7,764 (27.4)	240 (30.5)	1,585 (32.7)	
**Hospital admission in the last 30 days of life**					
No	18,967 (55.9)	15,994 (56.5)	439 (55.9)	2,534 (52.2)	<0.000
Yes	14,991 (44.1)	12,328 (43.5)	347 (44.1)	2,316 (47.8)	
**ED visit in the last 30 days of life**					
No	16,826 (49.5)	14,143 (49.9)	398 (50.6)	2,285 (47.1)	0.001
Yes	17,132 (50.5)	14,179 (50.1)	388 (49.4)	2,565 (52.9)	
**Secondary Outcomes**					
**Interventions in last 30 days of life**					
***Mechanical ventilation***					
No	33,256 (97.9)	27,767 (98.0)	768 (97.7)	4,721 (97.3)	0.006
Yes	702 (2.1)	555 (2.0)	18 (2.3)	129 (2.7)	
***CPR***					
No	33,800 (99.5)	28,208 (99.6)	786 (100.0)	4,806 (99.1)	<0.000
Yes	158 (0.5)	114 (0.4)	–	44 (0.9)	
***Defibrillation***					
No	33,915 (99.9)	*28,280−28,284	786 (100.0)	*4,845−4,849	0.513
Yes	43 (0.1)	*38-42	–	*1-5	
***Initiation of dialysis***					
No	33,763 (99.4)	28,173 (99.5)	780 (99.2)	4,810 (99.2)	0.031
Yes	195 (0.6)	149 (0.5)	6 (0.8)	40 (0.8)	
***PCI***					
No	33,950 (100.0)	*28,315−28,319	786 (100.0)	*4,845−4,849	0.634
Yes	8 (0.0)	*3-7	–	*1-5	
***Feeding tube***					
No	33,675 (99.2)	28,084 (99.2)	780 (99.2)	4,811 (99.2)	0.945
Yes	283 (0.8)	238 (0.8)	6 (0.8)	39 (0.8)	
***Blood transfusions***					
No	31,281 (92.1)	26,169 (92.4)	720 (91.6)	4,392 (90.6)	<0.000
Yes	2,677 (7.9)	2,153 (7.6)	66 (8.4)	458 (9.4)	
***Bronchoscopy***					
No	33,576 (98.9)	28,006 (98.9)	780 (99.2)	4,790 (98.8)	0.474
Yes	382 (1.1)	316 (1.1)	6 (0.8)	60 (1.2)	
**Chemotherapy in the last 14 days of life**					
No	32,760 (96.5)	27,295 (96.4)	762 (96.9)	4,703 (97.0)	0.089
Yes	1,198 (3.5)	1,027 (3.6)	24 (3.1)	147 (3.0)	
**ICU admission in the last 30 days of life**					
No	32,448 (95.6)	27,081 (95.6)	754 (95.9)	4,613 (95.1)	0.253
Yes	1,510 (4.4)	1,241 (4.4)	32 (4.1)	237 (4.9)	

**ED –** Emergency Department

**CPR –** Cardiopulmonary Resuscitation

**PCI –** Percutaneous Coronary Intervention

**ICU-** Intensive Care Unit

*Cell sizes with < 6 individuals (or if back calculation would lead to another cell size < 6) are suppressed as per ICES policy

#### Secondary outcomes.

Allophones were more likely to receive several aggressive interventions within the last 30 days of life compared to anglophones, including higher rates of mechanical ventilation, CPR, initiation of dialysis, and blood transfusion (**Table 2**). There were no significant differences in the receipt of chemotherapy in the last 14 days of life or ICU admissions in the last 30 days of life among the different linguistic groups.

After adjusting for multiple covariates detailed above, we found that compared to anglophones, allophones had significantly higher odds of death in hospital (odds ratio [OR] 1.35, 95% confidence interval [CI], 1.25–1.45), hospital admission (OR 1.16, 95% CI, 1.07–1.24) and an ED visit (OR 1.16, 95% CI, 1.08–1.24) in the last 30 days of life (**Table 3**). In contrast, while francophones had significantly increased odds of death in hospital (OR 1.23, 95% CI, 1.04–1.46) compared to anglophones, no significant difference was observed in the odds of hospitalization or ED visits in the adjusted analysis. Allophones had significantly increased odds of receiving several aggressive interventions within 30 days of death, including mechanical ventilation (OR 1.43, 95% CI, 1.16–1.76), CPR (OR 2.52, 95% CI, 1.73–3.69), initiation of dialysis (OR 1.56, 95% CI, 1.07–2.28), and blood transfusions (OR 1.23, 95% CI, 1.10–1.39). While francophones had increased odds of receiving blood transfusions (OR 1.34, 95% CI, 1.03–1.75), no significant association was observed for the receipt of other aggressive interventions. The adjusted analysis did not show significant differences among linguistic groups in ICU admissions in the last 30 days or chemotherapy administration in the last 14 days of life.

**Table 3:** Odd ratios of end-of-life outcomes across linguistic groups (anglophones as reference).

Outcomes	Francophone Crude OR (95% CI)	Francophone Adjusted OR* (95% CI)	Allophone Crude OR (95% CI)	Allophone Adjusted OR* (95% CI)
**Primary Outcomes**				
**Death in hospital**				
	**1.17 (1.00-1.36)**	**1.23 (1.04-1.46)**	**1.29 (1.21-1.38)**	**1.35 (1.25-1.45)**
**Hospital admission in the last 30 days of life**				
	1.03 (0.89-1.18)	1.10 (0.94-1.30)	**1.19 (1.12-1.27)**	**1.16 (1.07-1.24)**
**ED visit in the last 30 days of life**				
	0.97 (0.84-1.12)	1.05 (0.90-1.22)	**1.12 (1.06-1.19)**	**1.16 (1.08-1.24)**
**Secondary Outcomes**				
**Interventions in the last 30 days of life**				
***Mechanical ventilation***	1.18 (0.73-1.89)	1.35 (0.83-2.18)	**1.35 (1.11-1.64)**	**1.43 (1.16-1.76)**
***CPR***	–	–	**2.21 (1.56-3.15)**	**2.52 (1.73-3.69)**
***Defibrillation***	–	–	0.77 (0.30-1.95)	1.02 (0.39-2.67)
***Initiation of dialysis***	1.47 (0.65-3.32)	1.64 (0.71-3.80)	**1.58 (1.11-2.25)**	**1.56 (1.07-2.28)**
***PCI***	–	–	1.95 (0.39-9.64)	1.37 (0.26-7.19)
***Feeding tube***	0.91 (0.40-2.05)	1.09 (0.48-2.48)	0.96 (0.68-1.34)	1.01 (0.71-1.46)
***Transfusion***	1.13 (0.87-1.45)	**1.34 (1.03-1.75)**	**1.28 (1.15-1.42)**	**1.23 (1.10-1.39)**
***Bronchoscopy***	0.68 (0.30-1.54)	0.75 (0.33-1.71)	1.11 (0.84-1.47)	1.20 (0.88-1.62)
**Chemotherapy in the last 14 days of life**				
	0.85 (0.56-1.28)	1.19 (0.78-1.81)	0.84 (0.70-1.00)	1.14 (0.94-1.38)
**ICU admission in the last 30 days of life**				
	0.93 (0.65-1.33)	1.04 (0.72-1.51)	1.11 (0.96-1.28)	1.14 (0.98-1.34)

Note: Numbers highlighted in bold are statistically significant

*Adjusted for: age, sex, comorbidity count, neighbourhood income quintile, rurality, immigration status, type of cancer, ADL, CPS, CHESS score, and pain scale.

**Abbreviations: OR-** Odds ratio, **ED –** Emergency Department, **CPR –** Cardiopulmonary Resuscitation, **PCI –** Percutaneous Coronary Intervention, **ICU-** Intensive Care Unit

We also evaluated the receipt of palliative care in the last 180 days across linguistic groups (**Table 4**). Compared to anglophones and allophones, francophones were less likely to receive palliative care in the last 180 days prior to death (francophone: 80.4%, anglophone: 85.7%, allophone: 90.0%), and among those who had physician palliative care visits, the time from first visit to death was also shorter in francophones compared to anglophones (median in francophone: 60 days, anglophone: 88 days, allophone: 83 days). Allophones had the most overall physician-based palliative care visits in the last 180 days of life across all settings. Overall, the location of palliative care visits was primarily in complex care (long-term hospital stay) followed by outpatient.

**Table 4:** Distribution and type of palliative care visits in the last 180 days across linguistic groups.

	Total	Anglophone	Francophone	Allophone	P-value
	(n = 33,958)	(n = 28,322)	(n = 786)	(n = 4,850)	
**Time (days) from first physician palliative care visit to death**					
Mean (SD)	93.6 (66.6)	94.3 (66.6)	77.3 (65.6)	91.8 (66.0)	<0.001
Median (Q1-Q3)	87 (27-170)	88 (28-171)	60 (15-143)	83 (27-170)	<0.001
Missing (%)	13.8	14.3	19.6	10.0	
**Any physician palliative care, N (%)**					
No	4,695 (13.8)	4,056 (14.3)	154 (19.6)	485 (10.0)	<0.001
Yes	29,263 (86.2)	24,266 (85.7)	632 (80.4)	4,365 (90.0)	
**Number of physician palliative care visits**					
Mean (SD)	21.2 (22.6)	20.9 (22.1)	17.3 (21.1)	23.9 (25.4)	<0.001
Median (Q1-Q3)	15 (5-30)	14 (5-30)	10 (4-24)	17 (6-33)	<0.001
Missing (%)	13.8	14.3	19.6	10.0	
**Physician home/LTC palliative care visit, N (%)**					
No	19,178 (56.5)	16,017 (56.6)	519 (66.0)	2,642 (54.5)	<0.001
Yes	14,780 (43.5)	12,305 (43.4)	267 (34.0)	2,208 (45.5)	
**Number of physician home/LTC palliative care visits**					
Mean (SD)	2.3 (5.40)	2.2 (5.16)	2.3 (7.01)	2.4 (6.40)	0.105
Median (Q1-Q3)	0 (0-2)	0 (0-2)	0 (0-1)	0 (0-3)	<0.001
**Physician outpatient palliative care visit, N (%)**					
No	12,855 (37.9)	10,890 (38.5)	359 (45.7)	1,606 (33.1)	<0.001
Yes	21,103 (62.1)	17,432 (61.5)	427 (54.3)	3,244 (66.9)	
**Number of physician outpatient palliative care visits**					
Mean (SD)	3.8 (7.16)	3.7 (7.02)	2.7 (6.40)	4.5 (7.97)	<0.001
Median (Q1-Q3)	1 (0-4)	1 (0-4)	1 (0-2)	2 (0-6)	<0.001
**Physician inpatient palliative care visit, N (%)**					
No	21,617 (63.7)	18,559 (65.5)	483 (61.5)	2,575 (53.1)	<0.001
Yes	12,341 (36.3)	9,763 (34.5)	303 (38.5)	2,275 (46.9)	
**Number of physician inpatient palliative care visits**					
Mean (SD)	3.2 (10.60)	3.0 (10.03)	3.1 (8.98)	4.7 (13.53)	<0.001
Median (Q1-Q3)	0 (0-1)	0 (0-1)	0 (0-2)	0 (0-2)	<0.001
**Physician complex care of 3rd party palliative care, N (%)**					
No	10,181 (30.0)	8,804 (31.1)	318 (40.5)	1,059 (21.8)	<0.001
Yes	23,777 (70.0)	19,518 (68.9)	468 (59.5)	3,791 (78.2)	
**Time from first palliative home care visit to death (non-physician care)**					
Mean (SD)	15.7 (28.0)	15.7 (28.1)	17.8 (31.6)	15.2 (27.1)	0.306
Median (Q1-Q3)	4 (1–17)	4 (1–17)	5 (1–16)	3 (1–16)	0.712
Missing Data (%)	49.1%	48.3	59.8	52.0	
**Any palliative home care visit (non-physician care), N (%)**					
No	16,676 (49.1)	13,684 (48.3)	470 (59.8)	2,522 (52.0)	<0.001
Yes	17,282 (50.9)	14,638 (51.7)	316 (40.2)	2,328 (48.0)	
**Number of home care visits (non-physician care)**					
Mean (SD)	45.3 (46.9)	44.3 (46.1)	46.5 (49.0)	51.2 (50.7)	<0.001
Median (Q1-Q3)	28 (9-66)	27 (9-64)	28 (8-66)	32 (11-78)	<0.001
Missing Data (%)	49.1	48.3	59.8	52.0	
**Location of the first palliative care visit, N (%)**					
Complex care	14,759 (43.5)	12,191 (43.0)	275 (35.0)	2,293 (47.3)	<0.001
Home or LTC	4,145 (12.2)	3,381 (11.9)	97 (12.3)	667 (13.8)	
Inpatient	*2,939−2,943	*2,367−2,371	109 (13.9)	463 (9.5)	
Not specified	*1-5	*1-5	0 (0)	0 (0)	
No palliative care	4,695 (13.8)	4,056 (14.3)	154 (19.6)	485 (10.0)	
Outpatient	7,415 (21.8)	6,322 (22.3)	151 (19.2)	942 (19.4)	

**LTC** – Long Term Care

*Cell sizes with < 6 individuals (or if back calculation would lead to another cell size < 6) are suppressed as per ICES policy

## Discussion

In this population-based cohort study, we identified differences in the end-of-life outcomes in patients who died of cancer across linguistic groups. Both francophones and allophones exhibited 20–30% higher odds of death in hospital compared to anglophones, after adjusting for multiple covariates. In addition, allophones had 16% higher odds of hospital admission as well as ED visits within the last 30 days of life. Although infrequent, allophones were also more likely to receive multiple aggressive interventions in the last 30 days of life, including mechanical ventilation, CPR, initiation of dialysis, and transfusions.

Explanations for these findings are complex and multi-factorial. We hypothesize that a major factor is related to language barriers between patients and providers. Since all physicians and other healthcare practitioners in Ontario are required to speak English, the official language of the province, francophone and allophone patients may thus face significant language barriers. Studies have shown that language barriers can have significant negative impacts on access to and delivery of healthcare and perception of care and satisfaction, affecting patient outcomes [6–8]. Our previous work had shown that hospitalized francophones and allophones had lower rates of hospital harm, in-hospital mortality, and shorter length of stays when they received the majority of their care from a physician who spoke their primary language [2]. Similar benefits of language-concordant care are seen in outpatient settings, including long-term care and primary care [8–10]. For patients with cancer, given the complexity of care and issues requiring effective communication, language may play an even more crucial role. A lack of effective communication between patients and providers may lead to aggressive interventions being considered as the “default” approach to care, even at the end-of-life [11,12]. Future studies are needed to evaluate interventions that may improve language concordance and their impact on end-of-life outcomes. These include ensuring that language and language proficiency are an essential component of medical records, preferentially assigning staff who speaks patients’ primary language for the care of the patient, and making professional interpretive services readily available [13].

Another consideration is the impact of culture on treatment preferences at the end-of-life. Previous studies have shown that the impact of language was even more pronounced for racial and ethnic minorities who were also members of linguistic minority groups [6,7,13]. Factors such as sex or gender, race and ethnicity, in addition to culture, are often closely intertwined, and can influence treatment preferences and choices. For example, an Ontario study found that recent immigrants were more likely to die in the ICU compared to non-immigrants, and origin of birth, particularly among individuals born in South Asia, was associated with a greater risk of dying in ICU [14]. There could also be perceived stigma associated with certain care decisions, such as opting not to pursue aggressive interventions. In some countries where these patients were from, seeking care and dying in the hospital are perceived as the “norm” instead of dying at home [11]. Each of these factors could further complicate communication among patients, family, and health care teams, subsequently affecting health outcomes. Future research is needed to contextualize the quantity and impact of linguistic alignment on quality of care and end-of-life outcomes.

### Limitations

First, given the nature of retrospective study design, there is a risk of residual confounding despite consideration and adjustment for several covariates. Second, our study was limited to home care patients who had a completed RAI-HC assessment, as this remains the most reliable method to identify patient language in population-based studies. As such, the study population may limit the generalizability of our findings. However, as our target population is those with cancer within 6 months of death, a significant proportion of these patients would have needed support from home care, and conversely a large proportion of home care residents have cancer. Nevertheless, this population likely represents patients with more dependance. However, we believe that this is an important effort as a key contribution to start studying in this field. Additionally, given the limitation in data availability, we were unable to assess the use of interpreters (if any), or the language spoken by health care providers during encounters, as well as quality of palliative care services, which may have influenced end-of-life outcomes. Future studies incorporating more granular details on the use of interpreters or caregivers to overcome language barrier are needed. Lastly, we used ICD codes in the standardized algorithm to identify outcomes including the use of mechanical ventilation, CPR, initiation of dialysis, and more, that may be subject to coding errors.

## Conclusions

In Ontario, compared to anglophone patients, allophone home care recipients with terminal cancer had an increased risk of death in hospital, hospital admissions and ED visits, and were more likely to receive several aggressive interventions in the last 30 days of life, and francophones also had an increased risk of death in hospital. These findings contribute to a growing body of evidence highlighting language as an important determinant of health and a driver of inequities in end-of-life care. Addressing these disparities through health system planning and policy development is essential to promote health equity among linguistically diverse populations. Further research is warranted to explore the underlying drivers of these differences and to develop targeted strategies to improve outcomes for linguistic minority patients with cancer.

### Disclaimer

This study was based on data compiled by ICES. However, the analyses, conclusions, opinions, and statements expressed herein are those of the author(s), and not necessarily those of ICES.

This document used data adapted from the Statistics Canada Postal Code^OM^ Conversion File, which is based on data licensed from Canada Post Corporation, and/or data adapted from the Ontario Ministry of Health Postal Code Conversion File, which contains data copied under license from ©Canada Post Corporation and Statistics Canada. Parts of this material are based on data and/or information compiled and provided by Ontario Ministry of Health, CIHI, Ontario Health, Ontario Registrar General (ORG) – information on deaths, the original source of which is ServiceOntario, in addition to the Immigration, Refugees and Citizenship Canada (IRCC) current to March 2023, CIHI and the Ontario Ministry of Health. The analyses, conclusions, opinions, and statements expressed herein are solely those of the authors and do not reflect those of the data sources; no endorsement is intended or should be inferred.

## Supporting information

S1 Table List of interventions and billing codes. (DOCX)
