# Supplementary material for: The association between patient preferred language and end-of-life outcomes of home care patients who died from a cancer in Ontario, Canada – A retrospective cohort study
Source: PLoS One. 2026 Jun 22;21(6):e0351840. doi: 10.1371/journal.pone.0351840 (PMC13286143; doi:10.1371/journal.pone.0351840)
Supplement: S1 Table — (DOCX) [file pone.0351840.s001.docx]

Supplemental Table 1

| **Interventions** | **OHIP Billing Codes** | **CCI Codes/ICD-10-DAD** |
| --- | --- | --- |
| **Mechanical ventilation in last 30 days** | N/A | **Invasive:** 1GZ31CAEP, 1GZ31CAND, 1GZ31CAPK, 1GZ31CRND, 1GZ31GPND, 1GZ31JAGX  **Non-Invasive:** 1GZ31CAMP, 1GZ31CBEP, 1GZ31CBND, 1GZ31JAMD, 1GZ31JANC, 1GZ31JAPK  FLAG_MVENT_GE96, FLAG_MVENT_LT96 (DAD) |
| **Cardiopulmonary resuscitation (CPR) in the last 30 days** | N/A | 1.HZ.09.^^ |
| **Initiation of dialysis in the last 30 days** | N/A | 1.PZ.21  FLAG_DIALYSIS |
| **Percutaneous coronary intervention (PCI) in the last 30 days** | N/A | 1.IJ.50^^, 1.IJ.54, 1.IJ.57.GQ |
| **Feeding tube in the last 30 days** | J055, S118, Z532, S134, J063, Z540 | **Gastrostomy Feeding Tube:** 1NF53BABC, 1NF53BATS, 1NF53BTQB, 1NF53BTTS, 1NF53DAQB, 1NF53DATS, 1NF53HATS, 1NF53LAQB, 1NF53LATS, 522, 5510  **Gastrostomy-Jejunostomy Feeding Tube:** 1NK77EM, 1NK77RQ, 1NK53DATS, 1NK53LATS  FLAG_FEEDING_TB (DAD) |
| **Blood transfusion in the last 30 days** | N/A | 1.LZ.19  BTANY indicator (DAD) |
| **Bronchoscopy in the last 30 days** | Z327, Z360, Z342, Z359 | NEC 2.GM.70.^^ or 2GM70 |
| **Vasopressors (epinephrine) in the last 30 days** | N/A | 3E033XZ |
| **Chemotherapy in the last 2 weeks** | G345, G359, G381, G388, G382, G281 | Z511, Z512  FLAG_CHEMOTHER (DAD) |
| **Admission to ICU in the last 30 days** | N/A | SCU ADMDATE (DAD) |
